# Parkinson’s Disease and Risk of Fracture: A Meta-Analysis of Prospective Cohort Studies

**DOI:** 10.1371/journal.pone.0094379

**Published:** 2014-04-08

**Authors:** Li Tan, Ying Wang, Lingling Zhou, Yun Shi, Fan Zhang, Li Liu, Shaofa Nie

**Affiliations:** Department of Epidemiology and Biostatistics, School of Public Health, Tongji Medical College, Huazhong University of Science and Technology, Wuhan, P. R. China; University of Tampere, Finland

## Abstract

**Backgrounds/Objective:**

Parkinson’s disease (PD) is the second most common neurodegenerative disease among the elderly population. However, epidemiological evidence on the relationship of PD with risk of fracture has not been systematically assessed. Therefore, we performed this meta-analysis of prospective studies to explore the association between PD and risk of fracture.

**Methods:**

PubMed, Embase, Web of Science and Cochrane Library up to February 26, 2014 were searched to identify eligible studies. Random-effects model was used to pool the results.

**Results:**

Six studies that totally involved 69,387 participants were included for analysis. Overall, PD patients had an increased risk of fracture compared with control subjects (pooled hazard ratio = 2.66, 95% confidence interval: 2.10–3.36). No publication bias was observed across studies and the subgroup as well as sensitivity analysis suggested that the general results were robust.

**Conclusion:**

The present study suggested that PD is associated with an increased risk of fracture. However, given the limited number and moderate quality of included studies, well-designed prospective cohort studies are required to confirm the findings from this meta-analysis.

## Introduction

Parkinson’s disease (PD) is the second most common neurodegenerative disease among the elderly population. It is characterized clinically by three motor symptoms including resting tremors, rigidity and bradykinesia [1], [2], with an estimated prevalence of about 1% among people aged over 60 years old [3]. PD is still an incurable progressive neurological disease and seriously impairs the quality of life (QOL) [4]. Therefore, it is of great importance to improve QOL and functional capacity as well as reduce the risk of subsequent adverse results.

Previous studies have suggested that PD patients have an increased risk of falls and reduced bone mineral density (BMD) compared with healthy controls [5], [6]. However, both falls and lower BMD are recognized determinants for the increased risk for fractures [7]–[9]. In recent years, there is growing evidence suggesting an increased risk for developing fractures in patients with PD [6], [10]–[17]. Nevertheless, epidemiological evidence on the relationship of PD with fracture risk has not been systematically assessed. Since the strength of prospective studies is stronger than retrospective studies, we conducted a meta-analysis of prospective cohort studies to explore the effects of PD on incidence of fracture.

## Methods and Materials

### Search Strategy

We followed the Meta-Analysis of Observational Studies in Epidemiology guidelines [18] to conduct this meta-analysis. We undertook a systematic search on PubMed, EMBASE, Web of Science and the Cochrane library up to February 26, 2014 for relevant prospective cohort studies that reported the association between Parkinson disease and risk of fracture. We also scanned the reference lists of all retrieved articles to find if there were any additional literatures. The search terms used were ‘(Parkinson’s disease OR Parkinson disease) AND Fracture’. There was no language restriction.

### Study Selection Criteria

Studies were included if they met the following criteria: i.) had a prospective cohort design, ii.) evaluated the association between PD and at least one anatomical site of fracture, iii.) reported the relative risk (RR) or hazard ratio (HR) and its 95% confidence interval(CI), iv.) excluded participants with fractures at baseline. Studies that had retrospective design or did not report risk estimates or were conference abstract were excluded. Only the most recent study with the longest follow-up duration was included if publications were duplicated or originated from the same study population.

### Data Extraction and Quality Assessment

Data extraction was performed by two independent reviewers, with any discrepancies resolved by discussion. We extracted the following data from the included studies: name of the first author, year of publication, study location, age of the study population, ratio of males to females, follow-up years, outcome assessment method, total number of fracture cases and study population, site of fracture and the corresponding risk estimates with 95%CI, and confounders that were adjusted for in the analysis.

We used a 9-star system based on the Newcastle-Ottawa Scale (NOS) [19] to assess the study quality. The maximum score of 9 points could be assigned to a study which had the highest methodological quality, with 4, 2, 3 scores respectively being assigned to selection of study groups, comparability of study groups, assessment of outcomes and adequacy of follow-up. Studies with scores of 0–3, 4–6, 7–9 were respectively deemed as low, moderate, and high quality.

### Data Analysis

Data for any site of facture were included for main meta-analysis. However, for studies that only reported one specific site of fracture, the corresponding risk estimates with 95%CI were also included for analysis. In a study [6] which reported both hip fracture and non-spine, non-hip fracture in two separate analysis cohorts, only the HR regarding hip fracture was extracted for main analysis as hip fracture is the most devastating type of fracture [20]. We transformed HRs or RRs by taking their natural logarithms. Standard errors were calculated from ln (HR) or ln (RR) and corresponding CIs. Heterogeneity in results across studies was assessed using the Cochrane’s Q test and *I^2^* statistic [21], [22]. The heterogeneity was considered statistically significant when *P*≤0.10 and *I^2^*>50%. A random-effects model [23] was used to pool the results because this model takes into account both study sample size and between-study variation [24]. We also performed sensitivity analysis to explore the potential influence of each individual study on overall results. Subgroup analyses were carried out according to sex, study location, site of fracture, follow-up years, quality score and number of confounders for adjustment.

Publication bias was detected by using both Begg’s test [25] and Egger’s test [26]. Begg’s test or Egger’s test was considered to indicate significant statistical publication bias when *P*-value<0.05. All data analyses were conducted using STATA version 11.0 (Stata Corporation, College Station, TX).

## Results

### Literature Search

**Figure 1:**
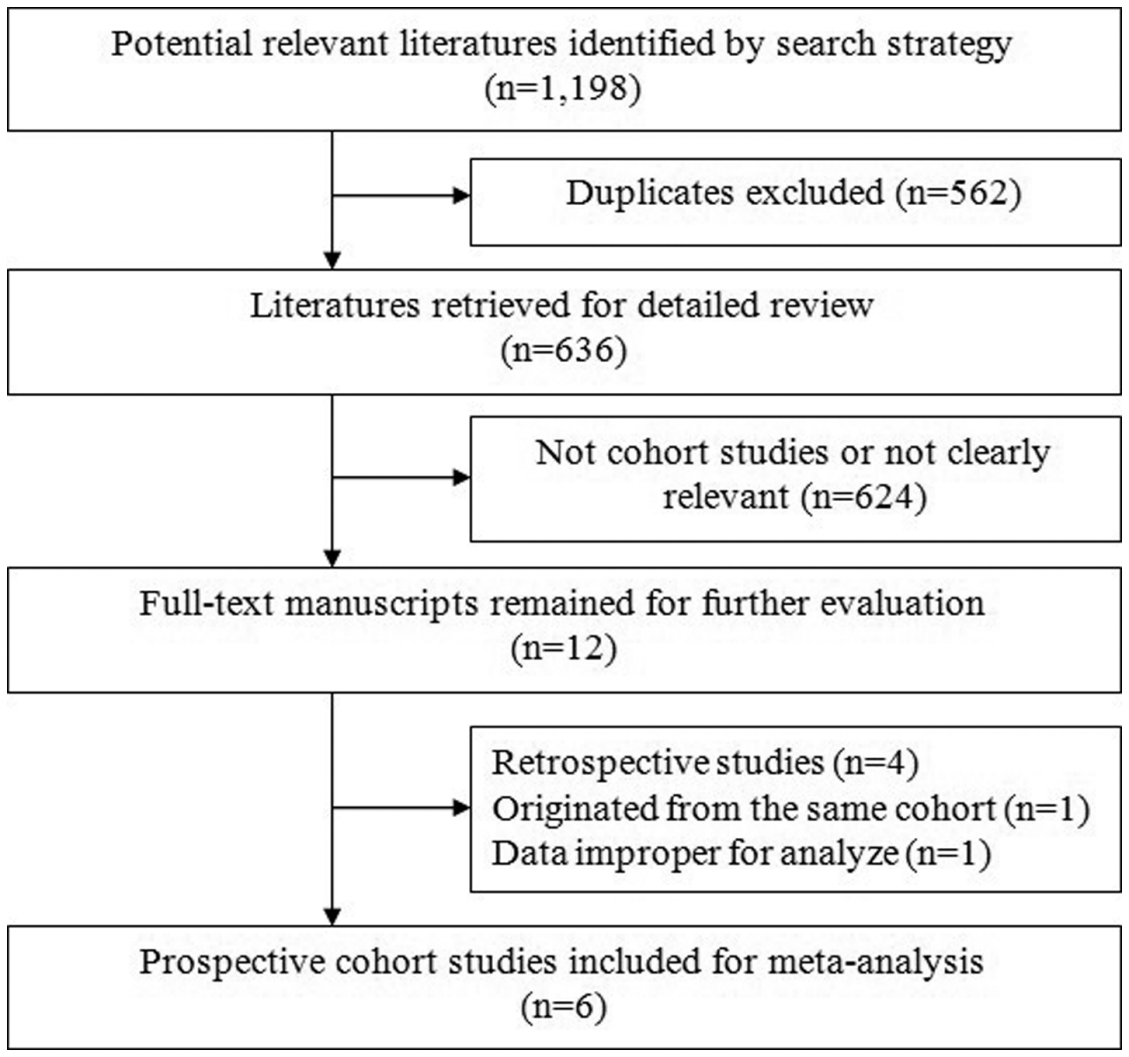
Selection flow of studies included in the meta-analysis. Two authors independently performed the literature search and selection to include prospective cohort studies regarding association between PD and risk of fracture, disagreement were resolved by discussion.

Figure 1 presents the study selection flow. A total of 1,198 articles were identified by the search strategy. After removing 562 duplicates, 636 articles were left for screening. After screening of titles or abstracts, 624 articles that were not clearly relevant or not cohort studies were excluded. After reading the full text of the remained 12 articles, 4 articles [13], [15], were excluded as they were retrospective studies, 1 article [11] was excluded as we included another article reported the same study with a longer follow-up duration. 1 study [28] calculated the odds ratio (OR) and its 95%CI based on an estimated number of people with PD using a crude PD prevalence from two studies. Because this kind of estimation failed to consider many potential confounding factors and thus the OR might not be accurate, thus we did not included this study. Finally, a total of 6 prospective cohort studies [6], [10], [12], [16], [29], [30] were included for meta-analysis.

### Study Characteristics

Table 1 summarizes the main characteristics of the studies for analysis. Of the 6 studies, 4 studies [6], [12], [16], [30] were conducted in the United States, 1 study [10] was conducted in Taiwan, while 1 study [29] was a conducted in 10 countries including Australia, Belgium, Canada, France, Germany, Italy, Netherlands, Spain, U.K., and U.S. 3 studies [6], [29], [30] only consisted of females, 1 study [12] only consisted of males while 2 studies [10],[16] involved both sex. The study population ranged from 392 to 43,832, with a total of 69,387 participants involved in. Adjusted HR was reported in 4 studies [6], [10], [12], [30]. The follow-up years were 3, 4.6, 8, 8.9, 9, and 13 respectively for the 6 studies. For the outcome assessment, 4 studies [6], [12], [29], [30] were based on self-report, while 1 [10] was ascertained by discharge diagnosis and 1 [16] was based on inpatient and outpatient records. Fractures were most frequently reported in the hip [6], [10], [16], [30]. Table 2 presents the results of quality assessment based on NOS, 4 studies were in high quality (1 [10] scored 9 and 3 [6], [16], [30] scored 7, respectively) while the other 2 studies [12], [29] were in moderate quality (scored 6 and 5 respectively).

**Table 1:** Characteristics of epidemiological studies of parkinson’s disease and risk of fracture included in the meta-analysis.

Studies	Studylocation	Ratio of Malesto Females, %	Age, years	Total cases/Total population	Follow-upyears	OutcomeAssessment	Site of fracture	Adjustment forConfounders
Taylor,2004 [30]	U.S.	0∶100	73.3±4.9^1^	604/6,787	8.9	Self-report andconfirmed byreview ofradiological reports	Hip	Age
Melton,2006 [16]	U.S.	61∶39	Median 71(range 40–97)	100/392	13	Inpatient andoutpatient records	Any/hip/spine/skull orface/pelvis/ribs/clavicle orscapula or sternum/distal forearm/other arm orhands/other leg orfeet	NA^2^
Schneider,2008 [6]	U.S.	0∶100	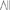 65	850/8,105	9(hip cohort); 8(non-spine,non-hip cohort)	Self-reported,confirmed byexpert review ofradiology reports	Hip/Non-spine and non-hip	Age, walking speed,depression, self-reportedhealth status, falls inpast year, weight change,and total hip bone mineraldensity
Fink,2008 [12]	U.S.	100∶0	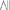 65	431/5,937	4.6	Self-report,confirmed bycentral review ofradiographic reports	Non-spine	Age
Chen,2012 [10]	Taiwan	49∶51	68.6±7.3^1^	201/4,334	8	Discharge diagnosis	Hip	Age, gender, state ofco-morbid hypertension,diabetes, diabetic neuropathy,osteoporosis, andhyperlipidemia
Gregson,2013 [29]	10 countries^3^	0∶100	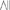 55	2,945/43,832	3	Self-report	Any	NA^2^

1 It was mean age ± standard deviation.

2 NA: Not available.

3 Australia, Belgium, Canada, France, Germany, Italy, Netherlands, Spain, U.K., and U.S.

**Table 2:** Methodological quality of cohort studies included in the meta-analysis.

Studies	Selection				Comparability	Outcome			Total scores
	Representativeness ofthe exposed cohort	Selection of theunexposed cohort	Ascertainmentof exposure	Outcome of interest notpresent at start of study	Control for importantfactor or additionalfactor^1^	Outcomeassessment	Follow-uplong enoughfor outcomesto occur	Adequacy offollow-up ofcohorts	
Taylor,2004 [30]	–	☆	☆	☆	☆☆	☆	☆	–	7
Melton,2006 [16]	☆	☆	☆	☆	–	☆	☆	☆	7
Schneider,2008 [6]	☆	☆	–	–	☆☆	☆	☆	☆	7
Fink,2008 [12]	☆	☆	–	–	☆☆	☆	☆	–	6
Chen,2012 [10]	☆	☆	☆	☆	☆☆	☆	☆	☆	9
Gregson,2013 [29]	☆	☆	☆	–	–	–	☆	☆	5

1 A maximum of 2 stars could be assigned to this item.

### Main Analysis

**Figure 2:**
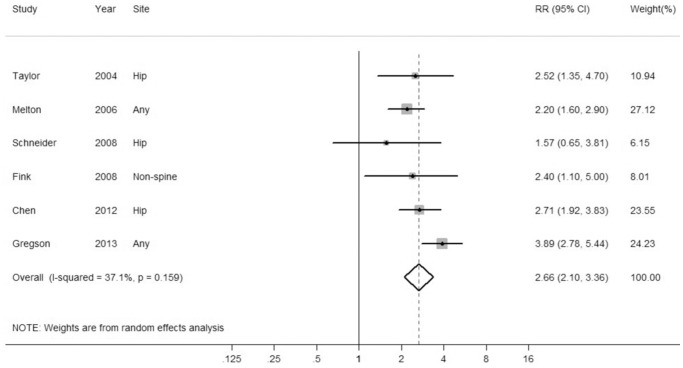
A forest plot of the association between PD and risk of fracture. Random-effects model was used to pool the overall hazard ratios (HRs) and 95% confidence intervals (CIs). The diamond represents the pooled HR and, the squares and the horizontal lines respectively represent the HR and 95% CI of each individual study.

Figure 2 shows a forest plot presenting individual and overall results. No statistically significant heterogeneity was observed across studies (*Pheterogeneity* = 0.159, *I^2^* = 37.1%). Overall, meta-analysis of the included studies by random-effects model suggested an increased risk of fracture in those with PD compared to those without PD (Overall HR = 2.66, 95% CI: 2.10–3.36).

### Subgroup and Sensitivity Analysis

Table 3 shows the effects of PD on fracture risk in subgroup analysis. From the results, we observed a similar summary HR in men (HR = 2.55, 95%CI: 1.77–3.67) and in women (HR = 2.54, 95%CI: 1.67–3.86), and greater summary HR in studies with a follow-up duration of less than 6 years (HR = 3.46, 95%CI: 2.30–5.19) than that of more than 6 years (HR = 2.36, 95%CI: 1.92–2.90). Subgroup analysis on other strata generally had similar results with the overall result.

**Table 3:** Summary risk estimates of the association between PD and risk of fracture^1^.

Factors	No. of studies	Summary adjusted HR (95%)	Heterogeneity *I* 2 (%)
Sex			
Male	2	2.55(1.77, 3.67)	0
Female	4	2.54(1.67, 3.86)	62.2
Study location			
US/Europe	5	2.60(1.89, 3.57)	49.7
Asia	1	2.71(1.92, 3.83)	NA
Site of fracture			
Any	2	2.91(1.66, 5.09)	83.9
Hip	4	2.66(2.07, 3.42)	0
Non-spine	2	1.61(0.70, 3.73)	51.8
Follow-up years			
>6	4	2.36(1.92, 2.90)	0
<6	2	3.46(2.30, 5.19)	23.5
No. of factors for adjustment			
>5	4	2.75(1.98, 3.83)	53.6
<5	2	2.42(1.56, 3.74)	21.3
Quality scroe			
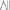 7	4	2.75(1.98, 3.83)	53.6
<7	2	2.42(1.56, 3.74)	21.3

1 PD: Parkinson’s disease.

2 HR: Hazard ratio.

For the result of sensitivity analysis, the pooled HR was not substantially influenced by any of the individual studies when omitting one study each time and recalculated the overall results, with a range from 2.36(95% CI: 1.94–2.88) to 2.87(95% CI: 2.22–3.73). As two included studies [16], [29] reported unadjusted HRs, we excluded them and re-pooled the result. However, the re-pooled HR was only slightly reduced and was similar to the general result (HR = 2.51, 95%CI: 1.92–3.27).

### Publication Bias

The Begg’s and Egger’s test suggested no evidence of publication bias in studies of PD and fracture risk (all *P*>0.05).

## Discussion

To our knowledge, this is the first meta-analysis of prospective cohort studies on PD and risk of fracture. Findings from the present analysis suggest that PD was associated with a 2.66-fold increased risk for fracture. Subgroup analysis and sensitivity analysis suggested that the overall result of this analysis was robust and there was no publication bias.

The underlying mechanism involved in the association between PD and fracture is still not clear. However, two main concerns might be explainable for their association. First, falls are important risk factors for fractures and 90% of fractures in older people result from a fall [13]. It was estimated that the annual incidence of falls among PD patients was 68%, with the majority of the fallers fell again in the following year [31]. Moreover, as the disease progresses, the occurrence of falls increased in PD patients [5]. Second, PD patients have lower BMD than healthy controls [32] and thus may have higher risk of fracture. In addition, low BMD in PD patients may be related to various factors, including reduced mobility, vitamin D deficiency, vitamin K deficiency, dysautonomia, and altered estrogen or growth hormone levels [33]. As all these factors are common in PD patients, they may act synergistically to the increased risk of fracture [34].

Along with the improvement and development of living conditions and medical care services, people tend to live longer compared with several decades ago, the global average life expectancy at birth increased from 64 years in 1990 to 70 years in 2011 according to WHO [35]. The estimated number of PD patients is expected to increase considerably because of the burden of chronic diseases being closely related to the increase of life expectancy in the most populous nations [36]. Meanwhile, the number of falls increases as the number of elderly population increases in many nations throughout the world [37]. Although it is not clear to what an extent the incidence of fractures were attributed to PD, the potential PD-related fracture and its corresponding burden of disease should not be neglected. Therefore, the findings from this study call for sufficient attention and care for the PD patients so as to reduce the subsequent fracture risk.

Both men and women have potential risk of fractures. In our study, we observed a similar summary HR in men (HR = 2.55, 95%CI: 1.77–3.67) and in women (HR = 2.54, 95%CI: 1.67–3.86). However, previous studies [38], [39] have suggested that postmenopausal women tend to have a higher fracture incidence than older men, which might be explained by the estrogen deficiency-related substantial decline in bone mass and changes in bone architecture [40]. The reason why men had similar pronounced risk might be related to the inconsistent conditions of individual studies, including age, site of fractures, progression stages of PD and ethnicity. Moreover, the stability of these findings is limited by the rare number of included studies.

Both severity and duration of PD are independent predictors of recurrent falls [41], [42], which implies that longer duration and more severity of PD may be associated with increased risk of fracture. However, in our study we found that the magnitude of increased risk in studies with shorter duration of follow-up was stronger than that with longer length of follow-up period (HR: 3.46 vs. 2.36). Nevertheless, considering the limited number of included studies, this finding should be interpreted with caution and further studies are required to determine the potential period for high risk of fracture in PD patients.

The present study has several strengths. Only prospective cohort studies were included for analysis, thus precluding the possibility of recall and selection biases. Meanwhile, no publication bias was observed, which suggests that no statistically significant effect of potential unpublished papers existed.

There are still several potential limitations. Firstly, while 4 included studies [6], [10], [12], [30] adjusted for several factors, 2 studies [16], [29] only reported unadjusted HRs. However, studies that reported unadjusted HR either used age as the time scale or were ordered by age in their estimation models, and the subgroup analysis excluding this two studies did not alter the general result, Nevertheless, the possibility that the association between PD and risk of fracture could be attributed to other potential confounders (such as vitamin D intake, dysautonomia, and growth hormone levels) could not be excluded. Secondly, although no statistical heterogeneity was observed between studies, there was potential clinical heterogeneity and methodological heterogeneity. However, results from sensitivity analysis and subgroup analysis suggested that the general result of this meta-analysis was rather robust. Thirdly, the information on fractures was self-reported without any objective ascertainments in one study [29], there was a possibility that it may lead to misclassification of fracture status due to potential recall bias. Nevertheless, the validity and accuracy of self-reported information for fractures were supported by previous studies [43], [44]. Finally, the length of follow-up varied among included studies, with 3 years in the shortest and 13 years in the longest; it is therefore difficult to determine the long-term impact of PD on fractures among the studies with insufficient follow-up duration.

## Conclusion

In conclusion, this meta-analysis indicates that PD is associated with an increased risk of fracture. However, given the limited number and quality of included studies, further well-designed studies are warranted to confirm the findings from our study.

## Supporting Information

Checklist S1 **PRISMA 2009 Checklist.**
(DOC)
